# Reviewed article: Campos HL, Fernandes GJA, Sousa DG, Conrado PLM, Campos EO, Santos Filho RAB, Costa PFF, Silva CM, Conrado GAM, Galvão PVM. Impacts of the COVID-19 pandemic on excess deaths: descriptive study, Pernambuco, 2020-2022. Epidemiol Serv Saude. 2025:34;e20240286

**DOI:** 10.1590/S2237-96222025v34e20240286.a

**Published:** 2025-09-01

**Authors:** Arthur Cruz

**Affiliations:** 1 Secretaria de Estado de Saúde de Mato Grosso do Sul, Campo Grande, MS, Brazil

## Peer review A

## Questionnaire

Does the manuscript contain new and significant information to justify publication? Yes

Does the Abstract (Summary) clearly and accurately describe the content of the article? Yes

Is the problem significant and concisely stated? Yes

Are the methods described comprehensively? Yes

Are the interpretations and conclusions justified by the results? Yes

Is adequate reference made to other work in the field? Yes

## Manuscript Structure

Length of article is: Adequate 

Number of tables is: Adequate 

Number of figures is: Adequate

Please state any conflict(s) of interest that you have in relation to the review of this paper: None

**Interest:** Good

**Quality**: Excellent

**Originality:** Good

**Overall:** Excellent

**Recommendation:** Minor Revision

## Comments

Item 1:

Nas linhas 16 e 17, o trecho a seguir está confuso:

As Regionais de Saúde e os períodos com maior excesso de óbitos foram aqueles em que ocorreram mais óbitos por COVID-19

A informação está redundante. Sugiro reescrever o trecho, sendo mais objetivo.

Item 2:

Na discussão, nas linhas 29, 32, 41, 46, 48 e 55 da página 4.

Verifique o uso de casas decimais em valores absolutos, como por exemplo em número de óbitos.

Rever em todo o texto

